# A meta‐analysis of the effectiveness and safety of endoscopic ultrasound‐guided choledochoduodenostomy employing electrocautery‐enhanced lumen‐apposing metal stents for biliary drainage after failed endoscopic retrograde cholangiopancreatography

**DOI:** 10.1002/deo2.70105

**Published:** 2025-04-23

**Authors:** Safiya Ibnawadh, Abdulrahman Alfadda, Abdulrahman Ibnawadh

**Affiliations:** ^1^ Department of Medicine Section of Gastroenterology King Faisal Specialist Hospital and Research Center Riyadh Saudi Arabia; ^2^ Department of Medicine King Faisal Specialist Hospital and Research Center Riyadh Saudi Arabia

**Keywords:** biliary drainage, choledochoduodenostomy, electrocautery‐enhanced lumen‐apposing metal stent, endoscopic retrograde cholangiopancreatography, endoscopic ultrasonography

## Abstract

**Objectives:**

Endoscopic ultrasound‐biliary drainage and choledochoduodenostomy (EUS‐CDD) are increasingly being used as alternative procedures for biliary drainage in patients in whom endoscopic retrograde cholangiopancreatography fails. Novel electrocautery‐enhanced lumen‐apposing metal stents (EC‐LAMS) are expected to be widely used for EUS‐CDD. We performed a systematic review and meta‐analysis to evaluate the technical and clinical success of EUS‐CDD using EC‐LAMS.

**Methods:**

We performed a comprehensive search of several databases from inception to May 2022 to search for relevant studies on the effectiveness and safety of endoscopic ultrasound‐guided biliary drainage using EC‐LAMS. The pooled rates of technical success, clinical success, and early and late adverse events were calculated.

**Results:**

Ten studies with a total of 481 patients were included in this analysis. The overall technical success rate was 94.3%, 95% confidence interval [CI] (91.5%–96.2%), The overall clinical success rate was 94.9%, 95% CI (92%–96.7%). The overall early adverse event rate was 5.1%, 95% CI (3.4%–7.8%), and the overall late adverse event rate was 10.8%, 95% CI (6.6%–17.2%).

**Conclusion:**

In patients with biliary obstruction with failed endoscopic retrograde cholangiopancreatography, EUS‐CDD using EC‐LAMS is a good alternate management option with a high success rate and relatively low adverse events.

## INTRODUCTION

Endoscopic retrograde cholangiopancreatography (ERCP) remains the gold standard first‐line treatment for biliary drainage in patients with biliary obstructions.^1^ Percutaneous transhepatic biliary drainage (PTBD) and surgical intervention are warranted when ERCP fails. However, both are associated with higher mortality and complication rates.^2^, ^3^ Recently, Endoscopic ultrasonography‐guided biliary drainage (EUS‐BD) has become an alternative option to PTBD and surgical intervention, and multiple randomized controlled trials (RCTs) and meta‐analyses have shown that EUS‐BD is comparable to PTBD. In a single‐center RCT and meta‐analysis, it was concluded that EUS‐BD is similar to PTBD in terms of clinical and technical success rates, as well as similar adverse event rates, with no statistical significance.^4^, ^5^ Another RCT and meta‐analysis concluded the same but with statistically significant lower adverse events and re‐intervention rates in the EUS‐BD group.^6^, ^7^

These variabilities in data can possibly be attributed to the different stents and techniques used for EUS‐BD. There is still no consensus on the best methods for EUS‐BD, and it entirely depends on the endoscopists' preferences and expertise. A recent meta‐analysis reported that choledochoduodenostomy (EUS‐CDD) has fewer adverse events than hepaticogastrostomy.^8^ Self‐expanded metal stents (SEMS) and lumen‐apposing metal stents (LAMS) are the most commonly used stents for EUS‐BD. A recent multicenter retrospective study compared fully covered SEMS to LAMS in biliary drainage and showed similar technical and clinical success, but with more delayed adverse events and re‐intervention rate in SEMS as it carries a higher chance of stent migration.^9^ A novel type of LAMS with a cautery‐enhanced tip (EC‐LAMS) is increasingly being used for biliary drainage; it has a simpler stent delivery system than LAMS and SEMS for EUS‐CDD. Individual studies have shown promising results, but no RCTs or meta‐analyses have evaluated the efficacy and safety of EC‐LAMS in EUS‐CDD.

In this meta‐analysis, we analyzed individual studies to examine the overall clinical and technical success and adverse events associated with EUS‐CDD using EC‐LAMS for biliary drainage in patients with failed ERCP.

## METHODS

This systematic review was conducted in accordance with the Preferred Reporting Items for Systematic Reviews and Meta‐Analysis guideline.^10^

### Search strategy

A comprehensive search of several databases and different keywords and Medical Subject Headings (MeSH) terms was used to search relevant data from various databases, including PubMed, MEDLINE, EMBASE, Scopus, ScienceDirect, and Cochrane Library from inception to May 2022. Additionally, we manually searched the references of the selected studies. The titles and abstracts of the selected studies were independently reviewed by two authors (Safiya Ibnawadh and Abdulrahman Ibnawadh). Further, search terms were combined using Boolean operators such as ‘AND’, ‘NOT’, and ‘OR’ to search for relevant studies regarding the effectiveness and safety of endoscopic ultrasound‐guided biliary drainage employing EC‐LAMS in terms of clinical and technical success. We included Studies published in English language or translated into English. Studies irrelevant to the research questions were excluded. A consensus was reached to overcome any differences in the article selection. The search strategy for PubMed is provided in Appendix 1.

### Selection criteria

Studies considered in this meta‐analysis met the following inclusion criteria: (1) patients with biliary obstruction for any cause, over 15 years old, and (2) had failed ERCP at least once and underwent choledochoduodenostomy (EUS‐CDD) using EC‐LAMS. We excluded studies (1) with populations younger than 15 years old or pregnant women; (2) where LAMS or other types of stents were used for EUS‐CDD; (3) studies in which EUS‐CDD was not used for employing EC‐LAMS; (4) with incomplete data or information or unclear descriptions of outcomes; (5) case reports, conference reports, animal studies, reviews, and letters; and (6) studies including patients with congenital biliary anomalies.

### Outcomes assessed

The primary outcome of this analysis was to calculate the pooled rate of overall technical success, which was defined as the successful placement of the transduodenal metal stent under EUS guidance with resultant biliary drainage. We calculated the pooled rate of overall clinical success, defined as the resolution of biliary obstruction by a 50% decrease in bilirubin level at four weeks.

The secondary outcome was early adverse events (procedural‐related and adverse events during hospitalization) and late adverse events (any adverse events that happened after discharge from the hospital).

### Data extraction

Selected studies that met the inclusion criteria were used for data extraction. The titles, abstracts, and full‐text papers were screened, and the extracted data were recorded in a standardized data extraction form. The following data were extracted.

· Study characteristics: authors, year of study, affiliation, country of origin, and study design
· Patient characteristics: sample size, reasons for failed ERCP, and etiology of bile duct obstruction
· Technical and clinical success rates.
· Early and late adverse events.

A consensus was reached to overcome any discrepancy in the data extraction.

### Quality assessment

Primary articles were reviewed, and the titles and abstracts of the studies were screened independently. Two reviewers independently appraised the full‐text articles of selected studies that met the inclusion criteria and discussed the appraisal results to reach a consensus. The risk of bias for the studies was assessed using a modified methodological index for non‐randomized studies (MINORS).^11^ The MINORS checklist uses a 3‐point scale to assess the quality of each item; each item was scored as 0 if not reported (high risk of bias), 1 if reported but inadequate (unclear risk of bias), and 2 if reported and adequate (low risk of bias), and the sum for each study was calculated. The maximum possible scores were 16 for non‐comparative studies and 24 for comparative studies. The first eight domains were used to evaluate the risk of bias in the non‐comparative studies included in this meta‐analysis. Studies with a score ≤11 were rated as low quality and studies with a score ≥12 as high quality. Two reviewers independently evaluated the potential risk of bias. The two reviewers reached a consensus when there was a difference in opinion on an item. If no agreement was reached, the independent opinion of a third reviewer was considered decisive.

### Statistical analysis

The primary outcome measure in this study was the efficacy of EC‐LAMS microspheres as assessed by the technical and clinical success rates. Early and late adverse events were the secondary outcome measures. Weighted pooled rates were calculated for the outcomes of interest with corresponding 95% confidence intervals (95% CIs). These were analyzed using a fixed‐effects model.^12^ Heterogeneity across the studies was assessed using Cochran's Q test and *I* ^2^ statistics. Cochran's Q test showed a *p*‐value <0.05, indicating the presence of heterogeneity. *I* ^2^, Unlike Q, does not inherently depend on the number of studies considered, with values of 25%, 50%, and 75% indicating low, moderate, and high levels of heterogeneity, respectively.^13^ A fixed‐effects design was used when *I* ^2^ < 50 % and *p* > 0.05; otherwise, a random‐effects model was used.^12^ A subgroup analysis was performed to investigate the cause of heterogeneity. Sensitivity analysis was also performed to evaluate the stability of the results. Egger's test, a weighted regression test, was conducted to evaluate publication bias. This was further assessed by visually examining the symmetry of the funnel plots.^14^ In all cases, statistical significance was set at *p* < 0.05. Statistical analysis was performed using Comprehensive Meta‐Analysis Version 3.

### Outcomes assessment

All the studies included in this meta‐analysis report treatment‐related mortality as a secondary safety outcome. The studies reported no deaths related to the procedures. However, it should be known that some of these studies did not actively monitor mortality so a lack of reported deaths may only be a reflection of the absence of reporting rather than the absence of death events.

## RESULTS

### Literature search

**FIGURE 1:**
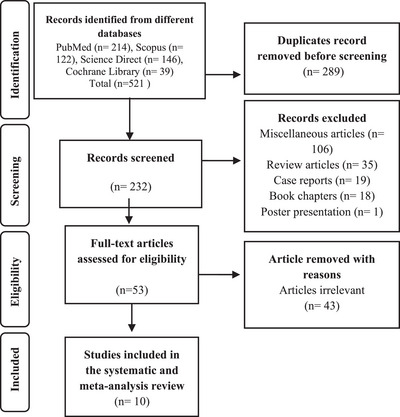
Flow chart of studies search and the articles selection process according to the Preferred Reporting Items for Systematic Reviews and Meta‐Analysis (PRISMA) guidelines.

The literature was searched using many specified electronic databases, such as PubMed, Scopus, ScienceDirect, and Cochrane Library, and manually through the references of peer‐reviewed journals, obtaining a total of 521 potential articles, 289 of which were removed because of duplication. After removing duplicates, 232 papers were evaluated for titles and abstracts, and 179 articles were excluded, including miscellaneous articles, review articles, and book chapters. The remaining 53 papers were examined entirely and 43 were eliminated based on reasons (Figure 1). This literature review included 10 studies published between 2016 and 2022.

### General characteristics

The characteristics and outcomes of the included studies are explained and summarized in Table 1; ten studies were included.^15^, ^16^, ^17^, ^18^, ^19^, ^20^, ^21^, ^22^, ^23^, ^24^ Three studies were reported from the USA,^16^, ^17^, ^19^ three from France,^18^, ^20^, ^24^ two from the UK,^22^, ^23^ and one each from Japan^15^ and Italy.^21^ All studies followed either a prospective or a retrospective design. The sample size ranged from to 16–120, reasons for failed ERCP before conducting EC‐LAMS were reported, and the most common cause was duodenal obstruction. The etiology of bile duct obstruction was also mentioned in detail. In addition, a 100% technical success rate was observed in three studies^15^, ^21^, ^22^ ^,^ and a 100% clinical success rate was observed in three studies.^19^, ^20^, ^21^ A mean follow‐up period of 30–188 days was reported in all studies.

**TABLE 1:** General characteristics of electrocautery‐enhanced lumen‐apposing metal stents (EC‐LAMS) included studies. (Early adverse events: Procedural‐associated adverse events or those occurring during hospitalization. Late adverse events are defined as adverse events that occur after hospital discharge.).

Study Id	Country	Study design	Sample size	Mean age (years)	Reason for failed ERCP	Bile duct etiology	Technical success	Clinical Success	Early adverse events	Late adverse events	Follow‐up
Tsuchiya et al.^15^	Japan and Hong Kong	Prospective	19	70.6	Duodenal stenosis, failed cannulation	Pancreatic cancers, cholangiocarcinoma, ampullary cancer, metastatic colon cancer, sarcoma, duodenal cancer, and metastatic gastric cancer	100%	95%	Not specified	36.80%	184 days
Anderloni et al.^16^	USA	Retrospective	16	69.6	Infiltration of the papilla by invasive cancer	Pancreatic cancer,	93.80%	93.30%	0.00%	6.60%	138.7 ± 124.6 days
						duodenal cancer, and					
						ampullary cancer					
Anderloni et al.^17^	USA	Retrospective	46	73.1	Tumoral involvement of papilla and cannulation failure	Pancreatic cancer and cholangiocarcinoma	93.50%	97.70%	0.00%	11.60%	114.37 days
Jacques et al.^18^	France	Retrospective	52	78	Inaccessible ampulla due to tumoral duodenal stenosis, prior duodenal stent placement, and duodenal stenosis	Distal pancreatic adenocarcinoma	88.50%	100.00%	3.80%	13.50%	157 days
el Chafic et al.^19^	USA	Retrospective	67	68.8 ± 11.8	Duodenal stenosis, failure to locate the papilla, or failed	Peri‐ampullary cancer and metastatic cancer	95.50%	100%	6.00%	17.15%	119 days
					biliary cannulation						
Jacques et al.^20^	France	Retrospective	70	75	Duodenal stenosis, tumoral infiltration of the papilla, and cannulation failure	Pancreatic adenocarcinoma, cholangiocarcinoma, duodenal lymphoma, and duodenal carcinoma	98.60%	98.60%	1.60%	10%	153 days
Tarantino et al.^21^	Italy	Prospective	21	Not mentioned	Tight stenosis of the CBD and non‐visualization of the ampulla of vater	Pancreatic head tumor, invasion of the papillary region, distal cholangiocarcinoma, and duodenum infiltration of unknown diagnosis	100.00%	100.00%	0.00%	14.28%	188 days
Ginestet et al., 2021	France	Retrospective	50	76.5	Duodenal stenosis, tumor invasion of papilla, and impassable stenosis	Pancreatic adenocarcinoma, distal colangiocarcinoma, and ampulla of vater carcinoma	97%	89%	4%	6%	118 days
Venkatachalapathy et al.^22^	UK	Prospective	20	76	NA	Locally advanced and metastatic pancreatic cancer, and primary duodenal tumor	100%	95.00%	5.00%	5.00%	30 days
On et al.^23^	UK	Retrospective	120	73	Inaccessible papilla, papillary distortion by tumor, and unsuccessful cannulation	Pancreatic ductal adenocarcinoma, duodenal adenocarcinoma, ampullary adenocarcinoma, distal cholangiocarcinoma, and metastatic disease from other primaries	90.80%	94.80%	7.50%	5.80%	70 days

### Quality assessment of included studies

Upon applying the MINORS methodological index for grading the 10 included articles, the highest score recorded was 14 and the lowest was 8. Among these studies, seven (70%) scored 12 or above and were rated as high quality, whereas three (30%) scored 11 or less and were rated as low quality. All studies lacked blinding in measurements and lacked proper sample size calculations, but most did not report any dropouts or changes in the follow‐up sample ratio. The summarized study quality assessment is presented in Table 2.

**TABLE 2:** Summary of the study quality assessment according to the modified methodological index for non‐randomized studies (MINORS) tool. Early adverse events: Procedural‐associated adverse events or those occurring during hospitalization. Late adverse events are defined as adverse events that occur after hospital discharge.

Study	A stated aim	Inclusion of consecutive patients	Prospective data collection	Endpoint appropriate to the study aim	Unbiased evaluation of endpoints	Follow‐up period appropriate to the major endpoint	Loss to follow‐up not exceeding 5%	Prospective calculation of sample size	Total score	Study quality
Tsuchiya et al.^15^	2	2	2	2	0	2	2	1	13	High
Anderloni et al.^16^	2	2	2	2	0	2	2	1	13	High
Anderloni et al.^17^	2	0	2	2	0	2	2	2	12	High
Jacques et al.^18^	2	0	0	2	0	2	2	2	10	Low
el Chafic et al.^19^	2	0	0	2	0	2	0	2	8	Low
Jacques et al.^20^	2	2	2	2	0	2	2	2	14	High
Tarantino et al.^21^	2	2	2	2	0	2	2	0	12	High
Ginestet et al., 2021	2	2	2	2	0	2	2	0	12	High
Venkatachalapathy et al.^22^	2	2	2	2	0	2	0	1	11	Low
On et al.^23^	2	0	2	2	0	2	2	2	12	High

### Primary outcomes

#### Overall technical efficacy

**FIGURE 2:**
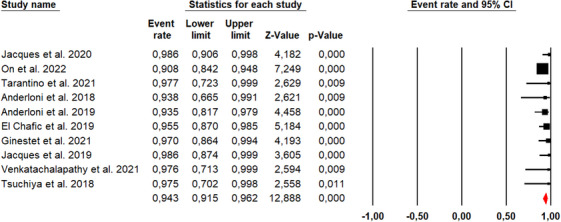
Forest plots for pooled technical success rates.

**FIGURE 3:**
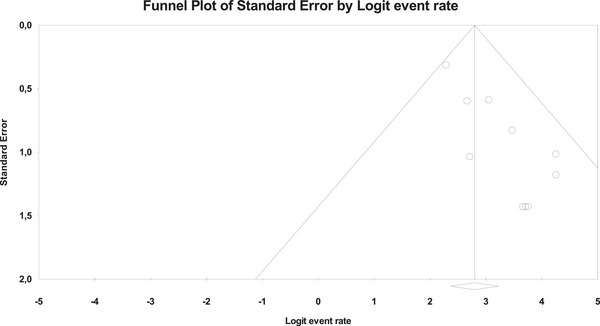
Funnel plot for publication bias of technical success rates.

Ten studies described the technical success rate of the EC‐LAMS technique, with an overall number of 481 patients (Figure 2). The overall technical success rate was 94.3% (95% CI, 91.5%–96.2%). Using Cochran's Q test and the *I* ^2^ statistic, we did not find significant heterogeneity (*Q*‐value = 8.307, *p* = 0.503, *I* ^2 ^= 0%). The funnel plot was asymmetrical (Figure 3), and Egger's test was statistically significant (*p* = 0.001).

### Clinical success rate

**FIGURE 4:**
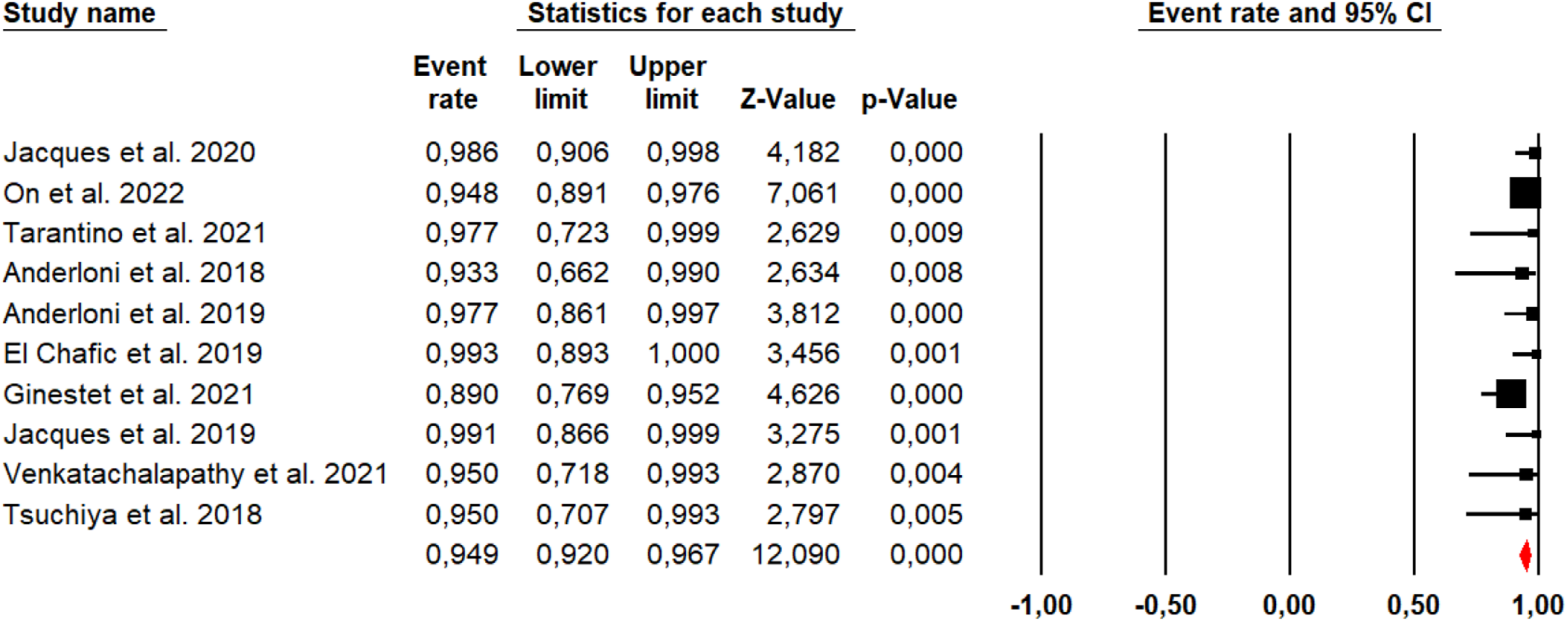
Forest plots for pooled clinical success rates.

**FIGURE 5:**
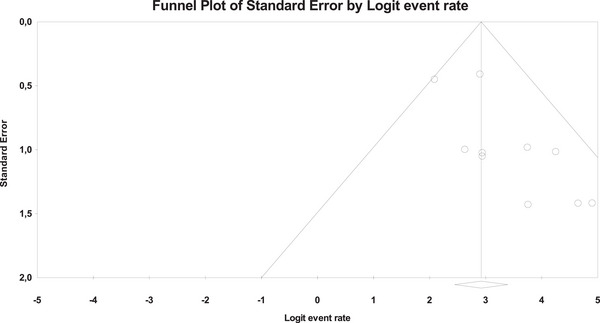
Funnel plot for publication bias of clinical success rates.

Ten studies described the clinical success rate of the EC‐LAMS technique, with an overall number of 481 patients (Figure 4). The overall clinical success rate was 94.9% (95% CI, 92%–96.7%). Using Cochran's Q test and the I^2^ statistic, we did not find significant heterogeneity (*Q*‐value = 9.673, *p* = 0.378, *I* ^2 ^= 6.96%). The funnel plot was asymmetrical (Figure 5), and Egger's test was statistically significant (*p* = 0.016).

### Early adverse events

**FIGURE 6:**
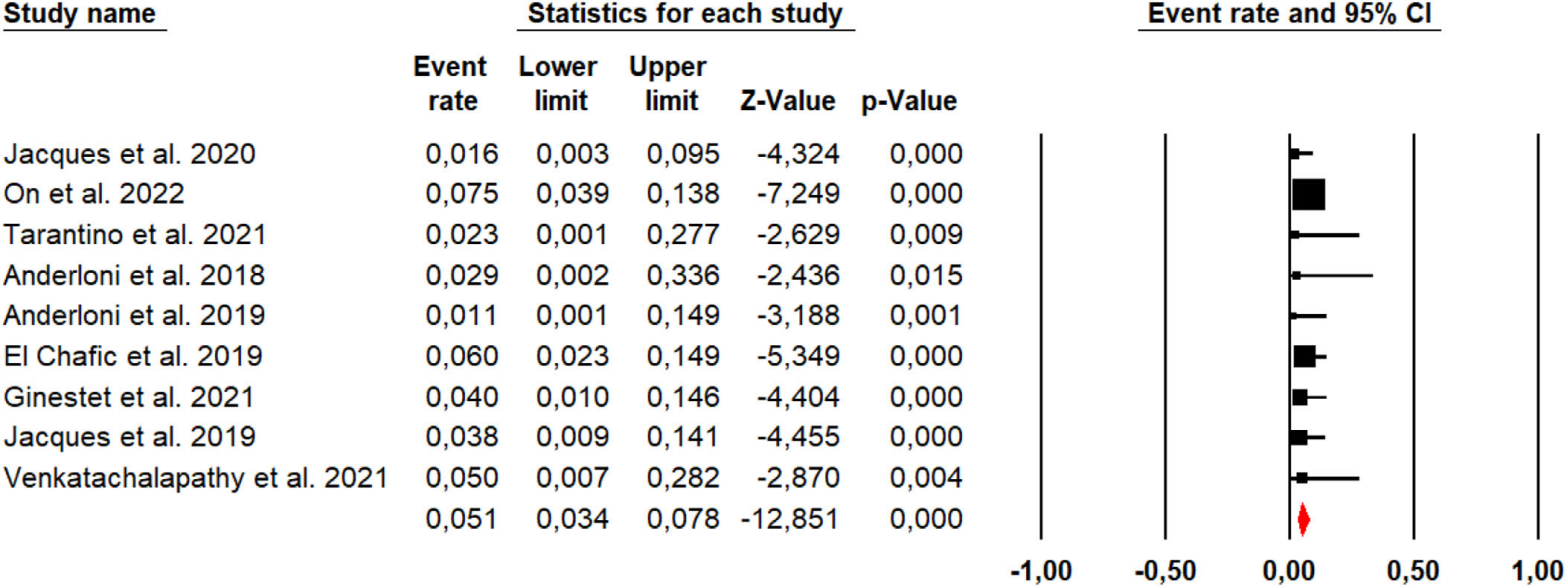
Forest plots for pooled early adverse event rates.

**FIGURE 7:**
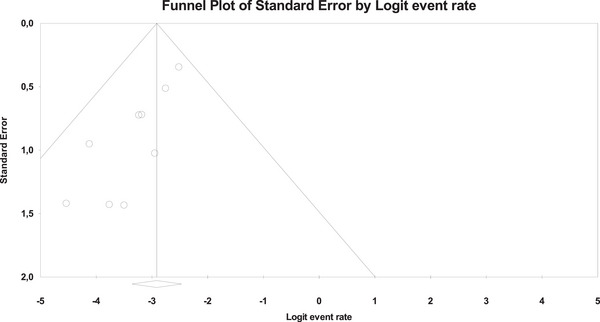
Funnel plot for publication bias of early adverse event rates.

Nine studies described the early adverse event rate of the EC‐LAMS technique, with an overall number of 462 patients (Figure 6). The overall early adverse event rate was 5.1% (95% CI, 3.4%–7.8%). Using Cochran's Q test and the *I* ^2^ statistic, we did not find significant heterogeneity (*Q*‐value = 5.181, *p* = 0.738, *I* ^2 ^= 0%). The funnel plot was asymmetrical (Figure 7), and Egger's test was statistically significant (*p* = 0.002).

### Late adverse events

**FIGURE 8:**
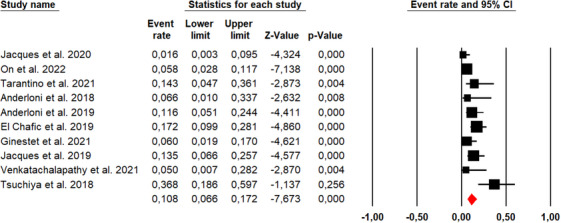
Forest plots for pooled late adverse event rate.

**FIGURE 9:**
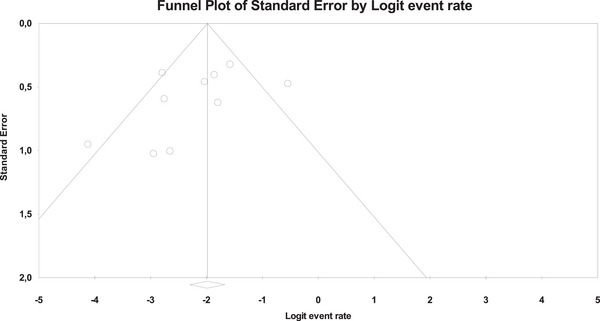
Funnel plot for publication bias of late adverse event rates.

Ten studies described the late adverse event rate of the EC‐LAMS technique, with an overall number of 481 patients (Figure 8). The overall late adverse event rate was 10.8% (95% CI, 6.6%–17.2%). Using Cochran's Q test and the *I* ^2^ statistic, we found significant heterogeneity among the included studies (*Q*‐value = 23.23, *p* = 0.006, *I* ^2 ^= 61.52%). The funnel plot appeared asymmetric (Figure 9); however, Egger's test was not statistically significant (*p*  =  0.20).

### Subgroups analysis

We performed a subgroup analysis of all outcomes (Table 3). The rates of technical success, clinical success, and early adverse events were not significantly different according to the study design, geographic origin of the studies, or sample size (*p* > 0.05), thereby confirming the absence of heterogeneity. However, the rate of late adverse events significantly differed between the studies for the three moderators (study design, continent, and sample size). When the study design was adopted as a moderator, the rate of late adverse events was significantly higher in the prospective studies (22.6%) than in the retrospective studies (10.2%; *p* = 0.018). Similarly, the rate of late adverse events was significantly different among the three continents (*p* = 0.001). Indeed, the highest event rate was observed in Asia (36.8%), followed by North America (14.4%) and Europe (7.9%). Similar results were found when sample size was used as a moderator. Indeed, the rate of late adverse events was significantly higher in studies with a sample size <30 (19.9%) than in studies with a sample size ≥30 (10.4%; *p* = 0.043). These findings indicate that the three moderators were sources of heterogeneity for late adverse events.

**TABLE 3:** Subgroup analyses.

	Technical success rate			Clinical success rate			Early adverse events rate			Late adverse events rate		
Subgroups	Studies	Event rate (95% CI)	Test for subgroup difference	Studies	Event rate (95% CI)	Test for subgroup difference	Studies	Event rate (95% CI)	Test for subgroup difference	Studies	Event rate (95% CI)	Test for subgroup difference
Study design												
Retrospective study	7	93.9% (90.8%–96%)	*p =* 0.251	7	94.7% (91.5%–96.8%)	*p =* 0.748	7	5.3% (3.4%–8.1%)	*p =* 0.702	7	10.2% (7.4%–13.9%)	*p =* 0.018^*^
Prospective study	3	97.6% (89%–99.5%)		3	95.8% (86.2%–98.8%)		2	3.8% (0.8%–17%)		3	22.6% (12.7%–36.9%)	
Continent												
Europe	6	94% (90.2%–96.3%)	*p =* 0.812	6	94.3% (90.7%–96.6%)	*p =* 0.566	6	5.3% (3.2%–8.5%)	*p =* 0.805	6	7.9% (5.3%–11.7%)	*p =* 0.001^*^
North America	3	94.5% (88.9%–97.4%)		3	97.2% (90.9%–99.2%)		3	4.7% (2%–10. %)		3	14.4% (9.2%–21.7%)	
Asia	1	97.5% (70.2%–99.8%)		1	95% (70.7%–99.3%)		0	NA		1	36.8% (18.6%–59.7%)	
Sample size												
<30	4	96.5% (88.7%–99%)	*p =* 0.385	4	95.1% (87%–98.3%)	*p =* 0.918	3	3.6% (0.9%–13.3%)	*p =* 0.582	4	19.9% (11.4%–32.4%)	*p =* 0.043^*^
≥30	6	93.9% (90.7%–96%)		6	94.8% (91.5%–96.6%)		6	5.4% (3.4%–8.3%)		6	10.4% (7.5%–14.1%)	

Early adverse events: Procedural‐associated adverse events or those occurring during hospitalization. Late adverse events are defined as adverse events that occur after hospital discharge.

### Sensitivity analysis

We performed sensitivity analysis and eliminated single studies to assess the reliability of the results. As shown in Table 4, the results suggest that no single study significantly affected the pooled results. Additionally, a stable trend in the event rate estimates was noted for the four outcomes, indicating that the meta‐analysis had strong reliability.

**TABLE 4:** Sensitivity analysis for meta‐analysis of technical success rate, clinical success rate, early adverse event rate, and late adverse event rate.

Outcome	Study excluded	Pooled event rate (95% CI)
Technical success rate	Tsuchiya et al.^15^	94.1% (95% CI: 91.3%–96.1%)
	Anderloni et al.^16^	94.3% (95% CI: 91.4%–96.2%)
	Anderloni et al.^17^	94.4% (95% CI: 91.4%–96.3%)
	Jacques et al.^18^	94% (95% CI: 91%–96%)
	el Chafic et al.^19^	94% (95% CI: 90.9%–96.1%)
	Jacques et al.^20^	93.9% (95% CI: 90.8%–95.9%)
	Tarantino et al.^21^	94.1% (95% CI: 91.2%–96.1%)
	Ginestet et al., 2021	94% (95% CI: 90.9%–96%)
	Venkatachalapathy et al.^22^	94.1% (95% CI: 91.3%–96.1%)
	On et al.^23^	96.3% (95% CI: 93.5%–97.9%)
Clinical success rate	Tsuchiya et al.^15^	94.9% (95% CI: 91.9%–96.8%)
	Anderloni et al.^16^	95% (95% CI: 92.1%–96.8%)
	Anderloni et al.^17^	94.6% (95% CI: 91.5%–96.6%)
	Jacques et al.^18^	94.6% (95% CI: 91.6%–96.6%)
	el Chafic et al.^19^	94.6% (95% CI: 91.5%–96.6%)
	Jacques et al.^20^	94.5% (95% CI: 91.3%–96.5%)
	Tarantino et al.^21^	94.8% (95% CI: 91.8%–96.7%)
	Ginestet et al., 2021	96.3% (95% CI: 93.7%–97.8%)
	Venkatachalapathy et al.^22^	94.9% (95% CI: 91.9%–96.8%)
	On et al.^23^	94.9% (95% CI: 91.2%–97.1%)
Early adverse event rate	Anderloni et al.^16^	5.2% (95% CI: 3.4%–7.9%)
	Anderloni et al.^17^	5.4% (95% CI: 3.5%–8.2%)
	Jacques et al.^18^	5.3% (95% CI: 3.4%–8.2%)
	el Chafic et al.^19^	5% (95% CI: 3.1%–7.9%)
	Jacques et al.^20^	5.5% (95% CI: 3.6%–8.4%)
	Tarantino et al.^21^	5.3% (95% CI: 3.4%–8%)
	Ginestet et al., 2021	5.3% (95% CI: 3.4%–8.2%)
	Venkatachalapathy et al.^22^	5.2% (95% CI: 3.3%–7.9%)
	On et al.^23^	3.9% (95% CI: 2.2%–6.7%)
Late adverse event rate	Tsuchiya et al.^15^	10.3% (95% CI: 7.6%–13.8%)
	Anderloni et al.^16^	12.3% (95% CI: 9.3%–16.1%)
	Anderloni et al.^17^	12.1% (95% CI: 9%–16.1%)
	Jacques et al.^18^	11.8% (95% CI: 8.7%–15.8%)
	el Chafic et al.^19^	10.8% (95% CI: 7.8%–14.7%)
	Jacques et al.^20^	12.7% (95% CI: 9.6%–16.7%)
	Tarantino et al.^21^	11.9% (95% CI: 8.9%–15.8%)
	Ginestet et al., 2021	12.7% (95% CI: 9.5%–16.7%)
	Venkatachalapathy et al.^22^	12.3% (95% CI: 9.3%–16.2%)
	On et al.^23^	13.9% (95% CI: 10.3%–18.5%)

Early adverse events: Procedural‐associated adverse events or those occurring during hospitalization. Late adverse events are defined as adverse events that occur after hospital discharge.

### Adverse events

The most common early adverse events were cholangitis (*n* = 8), followed by bleeding and perforation (*n* = 3) and abdominal pain (*n* = 2; Table 5).

**TABLE 5:** The most common adverse events.

Adverse events	Early adverse events (No. of patients)	Late adverse events (No. of patients)
Cholangitis	8	10
Bleeding	3	2
Perforation	3	0
Abdominal pain	2	0
Peritonitis	1	0
Stent occlusion	1	9
Recurrent biliary obstruction	0	10
Stent migration	0	4
Obstruction due to food impaction	0	3
Sump syndrome	0	2
Malignant obstruction	0	1
Obstruction in the second portion of the duodenum	0	2
Kinking	0	1
Suspected tumor ingrowth	0	1
Spontaneous dislodgement	0	1

Early adverse events: Procedural‐associated adverse events or those occurring during hospitalization. Late adverse events are defined as adverse events that occur after hospital discharge.

The most common late adverse events were cholangitis and recurrent biliary obstruction (*n* = 10), followed by stent occlusion (*n* = 9), stent migration (*n* = 4), and obstruction due to food impaction (*n* = 3).

## DISCUSSION

Biliary drainage using ERCP is the primary management option for patients with biliary obstruction of any cause.^1^ When failure occurs, other modalities to relieve obstruction are used, such as PTBD and EUS‐guided biliary drainage. Recent studies have shown that EUS‐BD has a better safety profile than that of PTBD.^3^, ^6^, ^7^ Due to two RCTs, EUS‐BD is being considered as an alternative for ERCP as a primary modality for biliary drainage.^24^, ^25^, ^26^ Based on a recent RCT, EUS‐CDD is expected to become more common for biliary drainage given its low adverse events compared to other approaches.^27^ Different stents can be used with EUS‐CDD, such as LAMS, SEMS, and plastic stents. A new type of LAMS that adopts an electrocautery‐enhanced tip is gaining popularity for EUS‐CDD, with promising results in individual studies and easy delivery systems.

The current meta‐analysis analyzed data on EUS‐CDD outcomes using EC‐LAMS. We included 10 studies with a total of 481 patients and analyzed the technical and clinical success rates as our primary outcomes, which were unified in definition between all included studies. Early and late adverse events were secondary outcomes.

In our analysis, the overall technical success rate of EC‐LAMS was 94.3%. In comparison, the clinical success rate was 94.9%, which was similar to that reported in a recently published meta‐analysis of EUS‐CDD using LAMS by Amato et al. This study included 820 patients, with a technical success rate of 94.8% and clinical success of 93.6%^28^.

In our analysis of adverse events, the calculated pooled rate of early adverse events was 5.1%. Late adverse events accounted for 10.8%, which is lower than the literature review of EUS‐CDD using LAMS by Jain et al., which included 92 patients and reported early adverse events of 10.8%, and 9.7% for late adverse events, respectively.^29^

We characterized the most common early and late adverse events. The most commonly encountered procedure‐related adverse event during hospitalization was cholangitis, which was reported in eight of 481 patients, followed by bleeding and perforation (3/481; Table 5).

As for late adverse events, the most common events were stent occlusion/biliary obstruction (10/481) and cholangitis (10/481). Compared to a meta‐analysis of adverse events of EUS‐CDD using LAMS that included 133 patients, the most common early adverse event was perforation (3.3%), followed by bleeding (2.3%) and cholangitis (1.6%), but they did not report late adverse events due to paucity of data in the included studies. However, their analysis reported stent obstruction in five of 133 patients.^30^

Our analysis of technical and clinical success and early adverse events was homogeneous, with the exception of late adverse events analysis, which showed moderate heterogeneity. The low sample size could explain it as was shown in our subgroup analysis using the sample size moderator, which confirmed a significant variation in results. Subgroup analysis also demonstrated that sample size was an important moderator, with studies with smaller sample sizes (<30) reporting a higher rate of late adverse events (19.9%) compared to larger studies (≥30; 10.4%). Proximity of study/diagnosis also created heterogeneity with a higher event rate seen in prospective studies as well as studies performed in Asia. Differences in study methodology, population characteristics, and regional practices may explain the findings, leading to a cautious interpretation of the results. In particular, studies conducted in Asia had a higher proportion of late adverse events than studies from other areas. These differences may reflect variations in procedural technique, demographics of the patients, or healthcare in these areas of the country. Such differences in training, stent delivery system, and post‐procedural care could explain these findings. And there are patient factors, such as comorbidities or access to follow‐up care, that may also factor in. Further studies are warranted to explore these regional differences to identify factors contributing to the rates of late adverse events.

The strength of our analysis is that it is the largest meta‐analysis to evaluate EUS‐CDD for biliary drainage using EC‐LAMS with well‐defined search criteria. In addition, the heterogeneity was low, and the sensitivity analysis of our data showed strong reliability. However, the limitation of this analysis is that it did not include RCTs, as none have been published yet. In addition, there was evidence of publication bias in our analysis; it is possible that studies with negative findings were likely not published contributing to publication bias. Thus, more RCTs with large sample sizes and multi‐center trials directly comparing EC‐LAMS and PTBD are still needed to evaluate the efficacy and safety of EUS‐CDD in biliary drainage using an EC‐LAMS. On the other hand, we recognize that shorter follow‐up periods in some studies could be seen as a potential limitation for an in‐depth assessment of mortality. That being said, the study of Venkatachalapathy et al. provided important data for the pooled analysis, but its limitations should be noted. In particular, the study had a limited follow‐up length of 30 days and a low MINORS score^22^. However, such an approach might be subject to various biases, especially regarding the reporting of late adverse events. To be sure, the lack of long‐term follow‐up and potential quality issues in the studies did call into question a somewhat less‐than‐expected sensitivity analysis, but those concerns were mitigated by the large number of studies contributing to the overall results.

In patients with biliary obstruction who fail ERCP, EUS‐CDD using EC‐LAMS is a good management option with a high success rate and relatively low complications. However, further studies and controlled trials are needed to compare the efficacy and complications of PTBD.

## CONFLICT OF INTEREST STATEMENT

None.

## ETHICS STATEMENT

Not Applicable.

## PATIENT CONSENT STATEMENT

Not Applicable.

## CLINICAL TRIAL REGISTRATION

Not Applicable.

## Supporting information



Study quality assessment
