# Supplementary material for: A meta‐analysis of the effectiveness and safety of endoscopic ultrasound‐guided choledochoduodenostomy employing electrocautery‐enhanced lumen‐apposing metal stents for biliary drainage after failed endoscopic retrograde cholangiopancreatography
Source: DEN Open. 2025 Apr 23;5(1):e70105. doi: 10.1002/deo2.70105 (PMC12018532; doi:10.1002/deo2.70105)
Supplement: Supplementary file 1 — Study quality assessment [file DEO2-5-e70105-s001.docx]

**Appendix 1:**

Study quality assessment.

| **Study Id** | **Year** | **Study design** | **Sample size** | **New Castle Ottawa scale** | | |
| --- | --- | --- | --- | --- | --- | --- |
|  |  |  |  | **Selection** | **Comparability** | **Outcome** |
| Tsuchiya et al | 2018 | Prospective | 19 | *** | ** | *** |
| Anderloni et al | 2018 | Retrospective | 16 | *** | ** | *** |
| Venkatachalapathy et al | 2021 | Prospective | 20 | *** | ** | *** |
| Anderloni et al | 2019 | Retrospective | 46 | *** | ** | *** |
| Jacques et al | 2019 | Retrospective | 52 | *** | ** | *** |
| El Chafic et al | 2019 | Retrospective | 67 | *** | ** | *** |
| Jacques et al | 2020 | Retrospective | 70 | *** | ** | *** |
| Tarantino et al | 2021 | Prospective | 21 | *** | ** | *** |
| Ginestet et al | 2021 | Retrospective | 50 | *** | ** | *** |
| On et al | 2022 | Retrospective | 120 | *** | * | *** |
